# Informed consent for clinical trials in acute coronary syndromes and stroke following the European Clinical Trials Directive: investigators' experiences and attitudes

**DOI:** 10.1186/1745-6215-9-45

**Published:** 2008-07-21

**Authors:** Piotr Iwanowski, Andrzej Budaj, Anna Członkowska, Wojciech Wąsek, Beata Kozłowska-Boszko, Urszula Olędzka, Wojciech Masełbas

**Affiliations:** 1 Association for Good Clinical Practice in Poland, ul. Postępu 18B (Neptun), 02-676 Warszawa, Poland; 2 International Centre for Therapeutic Research, Servier Poland, ul. Jana Kazimierza 10, 01-248 Warszawa, Poland; 3 Centre of Postgraduate Medical Education, Department of Cardiology, Grochowski Hospital, ul. Grenadierów 51/59, 04-073 Warszawa, Poland; 4 Institute of Psychiatry and Neurology, 2nd Department of Neurology, ul. Sobieskiego 9, 02-957 Warszawa, Poland; 5 Department of Experimental and Clinical Pharmacology, Medical University, Warsaw, ul. Krakowskie Przedmieście 26/28, 00-325 Warszawa, Poland; 6 Bayer HealthCare, Medical Department, Al. Jerozolimskie 158, 02-326 Warszawa, Poland; 7 PPD Development, ul. Postępu 18B (Orion), 02-676 Warszawa, Poland

## Abstract

**Background:**

During clinical trials in emergency medicine, providing appropriate oral and written information to a patient is usually a challenge. There is little published information regarding patients' opinions and competence to provide informed consent, nor on physicians' attitudes towards the process. We have investigated the problem of obtaining consent from patients in emergency-setting clinical trials (such as acute coronary syndromes (ACS) and stroke) from a physicians' perspective.

**Methods:**

A standardised anonymous 14-item questionnaire was distributed to Polish cardiac and stroke centres.

**Results:**

Two hundred and fourteen informative investigator responses were received. Of these investigators, 73.8% had experience with ACS and 25.2% had experience with acute stroke trials (and 1% with both fields). The complete model of informed consent (embracing all aspects required by Good Clinical Practice (GCP) and law) was used in 53.3% of cases in emergency settings, whereas the legal option of proxy consent was not used at all. While less than 15% of respondents considered written information to have been fully read by patients, 80.4% thought that the amount of information being given to emergency patients is too lengthy. Although there is no legal obligation, more than half of the investigators sought parallel consent (assent) from patients' relatives. Most investigators confirmed that they would adopt the model proposed by the GCP guidelines: abbreviated verbal and written consent in emergency conditions with obligatory "all-embracing" deferred consent to continue the trial once the patient is able to provide it. However, this model would not follow current Polish and European legislation.

**Conclusion:**

An update of national and European regulations is required to enable implementation of the emergency trial consent model referred to in GCP guidelines.

## Background

Despite tremendous advances in pharmacotherapy over recent decades, the expectations of both medical professionals and patients towards more effective and safer therapies are even greater. This is particularly true for treatment of vascular diseases such as acute coronary syndromes (ACS) and acute stroke, considering the numerous complications that can occur. Although treatments of ACS and stroke have improved greatly with thrombolytic and percutaneous coronary interventions, both conditions still have major therapeutic needs. To satisfy these needs, much research is being conducted by both academia and industry. However, in the era of the evidence-based medicine, novel therapies, even in emergency settings, must be accompanied by essential documentation of their pre-clinical and clinical efficacy and safety to be approved for general use. Clinical research nowadays is governed by widely accepted strict ethical and quality standards, based on the Declaration of Helsinki [1] and Good Clinical Practice (GCP) guidelines (Good Clinical Practice standards by the International Conference on Harmonisation, ICH, 1996, also introduced into general law in most European countries over recent years by the means of two European Community directives, 2001/20/EC and 2005/28/EC). Essential to these standards are the respect of autonomy of research participants and the importance of their well-being over the interests of science and society. Therefore one of the most fundamental principles of modern clinical research is the informed consent of participants. In the case of emergency trials, however, giving appropriate oral and written information to a prospective participant is usually a challenge for researchers and treating physicians. The informed consent process has to compete with the categorical need of starting necessary diagnostic procedures as quickly as possible, short therapeutic windows, and frequently impaired or absent patient's competence to give informed consent due to poor physical or mental state, shock, neurological deficit, or even unconsciousness [2,3]. Evidence exists that participation in acute coronary trials may delay the start of the reperfusion therapy[4], and at least a part of that delay might be due to a patients' involvement in the informed consent process. It has been recently demonstrated that allowing deferred consent for enrolment in a traumatic brain injury (TBI) trial reduces time to study drug administration by 50% compared to written proxy consent [5].

In the European countries, the ethical and legal environment for conducting biomedical research involving humans has dramatically changed over the last decade. This is due to the inflow of industry-sponsored GCP-compliant clinical trials, adoption of modern medical and pharma laws, the update of national and international ethical codes and conventions, and the implementation of relevant European Union directives. From the perspective of a Polish national, there are a number of conflicting statements in the national medical and pharma laws which have built up a nebulous environment for emergency research investigators. Specifically, these include a waiver from participant's consent, no regulations for a deferred consent, and restrictive regulations for proxy (surrogate) consent, as well as a remarkable tradition of involving patient's relatives in information and/or consent process.

Little is known about either a patients' competence to give consent, or their general opinion on emergency research [6,7]. A recent report, written following interview of a group of 12 American stroke patients, demonstrates patients' willingness to participate in emergency research, as well as acceptance of a waiver of consent [8]. Noteworthy evidence also exists that the public is aware of the importance of emergency research and that the normal rules for consent may not be easily applicable in these circumstances [9]. Likewise, little is known on physicians' attitudes, especially in terms of informed consent of research participants. Only anecdotal reports are available, such as that by Ågard et al. who investigated the attitudes of Swedish cardiologists to ACS research in 2004 [10], or a survey among European neuro-trauma centres on TBI trials in 2005 [11]. This has led us to investigate the problem of informed consent in emergency settings from the perspective of Polish physicians' who have frequently faced such issues in practice when conducting ACS or stroke trials.

## Methods

A standardised anonymous 14-item questionnaire (for detailed content see Table 1) was sent out in 2006 to all identified in-patient Polish cardiac and stroke centres (43 and 22, respectively). Ten copies were sent per centre. The questionnaires were accompanied by supporting letters from the heads of national reference centres for heart diseases and stroke (co-authors of this paper, A.B. and A.C., respectively). Stamped addressed envelopes were provided for easy response. Additionally, approximately 300 copies of the questionnaire were given for countrywide distribution and collection by clinical trial monitors, in person. The monitors were employed by 6 pharmaceutical company sponsors and contract research organisations who confirmed that they had conducted trials with ACS or recent stroke in Poland, and who agreed to voluntarily contribute to our research (see Acknowledgements). Again, stamped addressed envelopes were provided for easy response. Thirty-seven copies of the questionnaire were e-mailed to investigators conducting a large multicentre ACS trial (sponsored by Eli Lilly), and approximately 30 copies were personally distributed and completed at investigators' meetings organised by the sponsor of a stroke trial (Servier). In total, approximately 1000 copies of the questionnaire were distributed. In each case, clear instructions were provided to ensure that the questionnaire was only completed by physicians who had been personally involved in obtaining consent for administering a drug in an emergency clinical trial setting.

**Table 1:** Questionnaire items (in order of appearance) and respective percentage of answers obtained

	**Total (N = 214)**	Acute coronary syndrome subgroup (n = 158)*	Acute stroke subgroup (n = 54)*
What was the **scope **of the trial information and **how **was it delivered to trial participants?**			
**all-embracing **information, **verbal **+ written***	**53.3%**	47.5%	70.4%
**abbreviated **information, **verbal **and **written**	**38.8%**	41.8%	29.6%
**all-embracing verbal **+ **abbreviated written **information***	**15.0%**	14.6%	13.0%
**abbreviated verbal **+ **all-embracing written **information***	**10.7%**	12.0%	7.4%
**abbreviated verbal **information only	**4.2%**	4.4%	3.7%
**proxy **consent (by guardianship court) **only**	**0.0%**	0.0%	0.0%

Did you additionally seek **consent **of participants' **relative(s)**, if available?			
yes, always	**18.3%**	9.5%	44.4%
yes, sometimes	**43.7%**	41.4%	48.1%
rarely or exceptionally	**25.3%**	32.5%	5.6%
never	**12.7%**	16.6%	1.9%

**Following enrolment**, did you **inform **participants' **relative(s)**, if available, about their trial participation?			
yes, always	**54.2%**	43.0%	87.0%
yes, sometimes	**33.7%**	41.8%	11.1%
rarely or exceptionally	**9.8%**	12.7%	1.9%
never	**2.3%**	2.5%	0.0%

Did the involvement of a participant's **relative(s) influence **the **time **necessary to obtain informed consent?			
yes, delayed	**55.6%**	55.1%	57.4%
yes, shortened	**6.6%**	6.3%	7.4%
no influence	**29.4%**	27.8%	35.2%
not applicable (no involvement)	**8.4%**	10.8%	0.0%

How do patients **react **to a trial proposal in an emergency condition?			
positively/somewhat positively	**65.7%**	61.1%	77.8%
equal proportions for positive and negative responses	**29.1%**	33.8%	16.7%
negatively/somewhat negatively	**1.0%**	1.3%	0.0%
uncertain	**4.2%**	3.8%	5.5%

Is an emergency (conscious) patient **able **to **understand **the **nature **of the trial and consciously **decide **whether or not to participate?			
always/most often	**32.3%**	31.7%	33.3%
some patients are able	**46.7%**	43.0%	59.3%
no or few patients are able	**17.3%**	20.9%	5.6%
uncertain	**3.7%**	4.4%	1.8%

How much of the **verbal **information received by the patient is really **understood**?			
all/almost all	**32.9%**	33.8%	31.5%
some	**58.2%**	58.6%	55.5%
almost none	**1.9%**	2.5%	0.0%
uncertain	**7.0%**	5.1%	13.0%

How much of the **written **trial **information **does an emergency patient **actually read**?			
all/almost all	**14.5%**	13.3%	18.5%
some	**62.1%**	62.0%	61.1%
almost none	**19.2%**	21.5%	13.0%
uncertain	**4.2%**	3.2%	7.4%

The **amount of information **supposed to be given to patient was, in general:			
too comprehensive	**80.4%**	85.4%	64.8%
adequate in regard to the patient's condition	**17.7%**	13.3%	31.5%
too brief	**0.0%**	0.0%	0.0%
uncertain	**1.9%**	1.3%	3.7%

How does a trial proposal to an emergency patient affect their **trust **in the physician?			
increases trust	**26.3%**	27.4%	24.1%
neither increases nor decreases trust	**48.3%**	49.0%	46.3%
decreases trust	**9.9%**	10.2%	9.2%
uncertain	**15.5%**	13.4%	20.4%

Does **informing **a participant's **relative(s) **about their trial participation **make sense **at all?			
yes	**68.1%**	63.3%	83.0%
no	**12.7%**	14.6%	5.7%
uncertain	**19.2%**	22.1%	11.3%

Which of the following **models **of informed consent in emergency settings would be **the best**?			
**all-embracing **information (like non-emergency trials), **verbal **and written***	**14.0%**	11.4%	22.2%
**abbreviated **information, **verbal and written **+ **abbreviated consent form**, with obligatory **all-embracing **written consent **to continue **the trial once the participant's status has sufficiently **improved **(ICH GCP-based)***	**78.0%**	81.7%	66.7%
**abbreviated oral **information + only **verbal consent**, with obligatory **all-embracing **written consent **to continue **the trial once the participant's status has sufficiently **improved **(currently unrealistic under national regulations)***	**7.5%**	6.3%	11.1%
other model	**0.5%**	0.6%	0.0%

* two respondents declaring experience with both acute coronary syndrome and stroke trials were not taken into account for subgroup analysis

** multiple choice possible; all other items were single choice

*** embracing all aspects required by Good Clinical Practice (GCP) and law for a regular trial

## Results

Two-hundred and eighteen completed questionnaires were returned (213 paper and 5 electronic copies). We considered this amount to be representative for the target group of physicians, taking into account the number of cardiologists and neurologists in Poland (approx. 1400 and 2000, respectively, plus presumably several hundreds of young physicians with ongoing specialisation or residency in those fields). We also considered the fact that only a proportion of them work in inpatient cardiac and stroke centres (no detailed national data on how many; presumably approx. 1/4 of each population), of which only a proportion have conducted emergency clinical trials. Informative responses were 97.2 – 100.0% per question. Four questionnaires were excluded from further analysis as no experience with ACS or stroke trials was reported (other emergency conditions only). One hundred and fifty eight responders (73.8%) declared experience with obtaining informed consent in ACS trials (of these 67.7% were trials sponsored by industry, 1.3% were academic, 27.8% were involved in both academia and industry-sponsored trials, and 3.2% gave no answer). Fifty-four responders (25.2%) had experience with acute stroke (of these 61.1% were in trials sponsored by industry, 13.0% were academic, 24.1% were involved in both academia and industry-sponsored trials, and 1.8% gave no answer). Two responders (1.0%) had experience with both ACS and acute stroke trials: one through clinical trials sponsored by industry and one through both industry and academia. In addition, 26 responders from both groups have also reported experience with informed consent processes in other emergency therapeutic fields (arrhythmia, acute heart failure, injury, epilepsy and migraine).

Each component of the questionnaire, and the corresponding percentage of informative answers obtained, is presented in Table 1.

## Discussion and conclusion

Our results show a lack of consistency in current practice for delivering trial-related information to ACS and stroke patients. The most commonly delivered consent model is based on complete written and verbal information ("all-embracing" in terms of the 20 items defined in ICH GCP, possibly extended by national ethical and legal standards). This consent methodology is even more frequent in acute stroke than ACS trials. Even though a legal option based on proxy consent (by guardianship court in case of Poland) exists for performing trials in acutely incompetent patients, it was not used by any of our respondents. In our opinion, this finding demonstrates the inadequacy of involvement of external bodies for proxy consent in emergency settings, at least with the current model of their functioning in practice.

Investigators' perceptions of patients' reactions to emergency trials were predominantly positive (more pronounced in acute stroke subgroup). Investigators generally did not feel that they had compromised their patients trust. However, most patients were regarded to be only partially able to understand the nature of the trial. While more than 90% of investigators think verbal communication is entirely or partially understood by emergency patients, less than 15% of respondents consider that written information is fully read by patients, and as many as 19.2% (even more in the ACS subgroup) observe that almost none of the written information is read. This corresponds with results available from literature on the limited competence of acute coronary patients, as well as stroke patients, to give informed consent [12-15]. In particular, the low rate of patients having read the information sheet, as perceived by our respondents, correlates well with the percentage of patients who admitted to having not read the information sheet (25%) in a recent ACS trial DANAMI-2 [16]. The vast majority of investigators (especially ACS trialists) consider the amount of information given to emergency patients to be too lengthy. This finding supports the pioneer research by Mader and Playe in late 1990's who concluded that informed consent forms used in emergency medicine research may be too complex for the average patient to understand [17]. These findings do however need verification by studies directly looking at emergency patients' perception and their actual understanding of trial information. Such research is sparse, in particular in the European setting [18].

Although no formal requirement exists (neither legal nor stated in the national Medical Code of Ethics), more than 50% of investigators (and almost all acute stroke trialists) sought, more or less frequently, parallel consent (assent) of patients' relatives (if available), even if it increased the time necessary to obtain consent. This supports the study by Kane et al. who found that obtaining assent from a patient's relative was the most frequently chosen option by investigators of a recent international stroke trial [19]. This also corresponds with the results of the study by Kompanje et al. among neuro-trauma physicians, 48% of whom considered patients relatives, in turn, as unable to make a balanced decision towards treatment [11]. Furthermore, nearly 90% of investigators in our survey (and almost all acute stroke trialists) informed relatives of trial participants of their actual participation. Our data show that involvement of emergency patients' relatives in the consent process, at least in the form of delivering information on trial participation, should be considered when updating ethical codes and legal regulations.

The last point on the questionnaire tried to establish which was the preferrential model for obtaining informed consent in emergency settings. The response to this question was strikingly repetitive. As many as 78% of investigators (even more in the ACS subgroup) favoured the two-step model proposed by the ICH GCP guidelines: abbreviated verbal and written consent in emergency conditions along with obligatory "all-embracing" deferred consent to continue the trial once the participants' status has sufficiently improved. This is in line with other evidence collected, where psychological and sociological methods demonstrate that a simplified informed consent procedure in an emergency setting may result in better understanding and retention of information by participants [18]. Together with our results, this supports the concept of simplified consent under emergency conditions and makes the deferral of complete consent ethically defendable, since the real understanding and retention of information by participants constitutes the principal aspect of *informed *consenting. However, this model conflicts with the current European legislation (requiring either "all-embracing" verbal and written information in order to obtain the patient's own consent or, alternatively, absolute proxy consent), as regulated by the Directive 2001/20/EC and implemented respectively to national laws, including Polish. This inadequacy of European legislation towards emergency research has already been highlighted by other authors [20,21], in contrast to the modern US regulations on emergency research allowing a waiver from consent [22]. We believe that our data supports the need to update the European regulations by building on the model of emergency trial consent found in ICH GCP guidelines.

## Competing interests

The authors declare that they have no competing interests.

## Authors' contributions

PSI conceived the study. All authors participated in the design of the study and formulated the study questionnaire. AB and AC identified the target cardiac and stroke centres and coordinated sending of questionnaires directly to responders. PSI, BKB, WM and UO coordinated distribution, completion and return of questionnaires via the trial monitors. PSI performed the analysis of results and drafted the manuscript. AB, AC, WW, UO and WM helped to finalise the manuscript. All authors read and approved the final manuscript.
